# Pulmonary artery coil embolisation prevented tumour progression in a patient with advanced squamous cell lung carcinoma

**DOI:** 10.1080/03009734.2020.1753863

**Published:** 2020-04-30

**Authors:** Virginija Šileikienė, Viktorija Gurskytė, Ingrida Zeleckienė, Elena Bernotienė, Sigitas Čibiras

**Affiliations:** a Clinic of Chest Diseases, Immunology and Allergology, Institute of Clinical Medicine, Faculty of Medicine, Vilnius University, Vilnius, Lithuania;; b Faculty of Medicine, Vilnius University, Vilnius, Lithuania;; c Centre of Radiology and Nuclear Medicine, Vilnius University Hospital Santaros Klinikos, Vilnius, Lithuania;; d Centre of Cardiology and Angiology, Vilnius University Hospital Santaros Klinikos, Vilnius, Lithuania

**Keywords:** Alternative therapy, coil embolisation, non-small cell lung cancer, spontaneous regression, squamous cell carcinoma

## Abstract

**Background:** Squamous cell lung carcinoma (SqCLC) is a type of non-small-cell lung cancer, accounting for 25–30% of all lung cancer cases with a median advanced stage survival of 8–11 months. Here we present a rare case of long-term survival with metastatic SqCLC following coil embolisation of the right pulmonary artery.

**Case presentation:** The 49-year-old patient was diagnosed with stage IV (cT4N3M1) SqCLC in 2007 due to a biopsy-proven central malignant tumour in the right lung and bilateral mediastinal lymphadenopathy. A magnetic resonance imaging scan also revealed a metastatic lesion in the liver. Soon after the diagnosis, the patient experienced pulmonary haemorrhage, which was managed by obturating the intermediate bronchus and performing coil embolisation of the right pulmonary artery. The patient also received chemotherapy in 2007 and 2009 without radiological changes. At three different time points in years 2010–2019, biopsies of the primary tumour were taken. All showed dense connective tissue with no indication of cancer growth. In 2020, a positron emission tomography scan showed no pathological metabolic activity in the lungs and liver. Currently, the patient remains in a stable clinical condition with a good performance status.

**Conclusion:** The long-term clinical benefit indicates a direct effect of coil embolisation on tumour progression. We suggest that coil embolisation of tumour-feeding arteries could be considered as a potential treatment method for patients with SqCLC.

## Introduction

Lung cancer remains the leading cause of malignancy-related mortality worldwide, accounting for approximately 25% of all cancer deaths (1,2). One of the most prevalent histological types of lung cancer is squamous cell lung carcinoma (SqCLC), which constitutes 25–30% of all lung cancer cases and is strongly associated with cigarette smoking (3,4). The survival rates of lung cancer are largely dependent on the cancer stage at the time of diagnosing and the histological type of the tumour. On average, 50.3% of patients with localised stage lung cancer survive for 5 years (2). Unfortunately, 48.7–57% of non-small cell lung cancer (NSCLC) patients are diagnosed in late stages of the disease, when the options for treatment are limited and the chances of surviving are poor (2,5,6). For instance, the median survival of patients with advanced SqCLC receiving first-line platinum-based chemotherapy is only 8–11 months (7). Nevertheless, there have been nearly 40 cases of spontaneous regression and longer survival in patients with advanced NSCLC documented in literature from 1950 to 2020, most of whom were diagnosed with SqCLC (8,9). In this paper, we present a rare case of long-term survival with metastatic SqCLC following chemotherapy and endovascular embolisation of the right pulmonary artery with 33 coils due to massive haemoptysis.

## Case presentation

**Figure 1.**
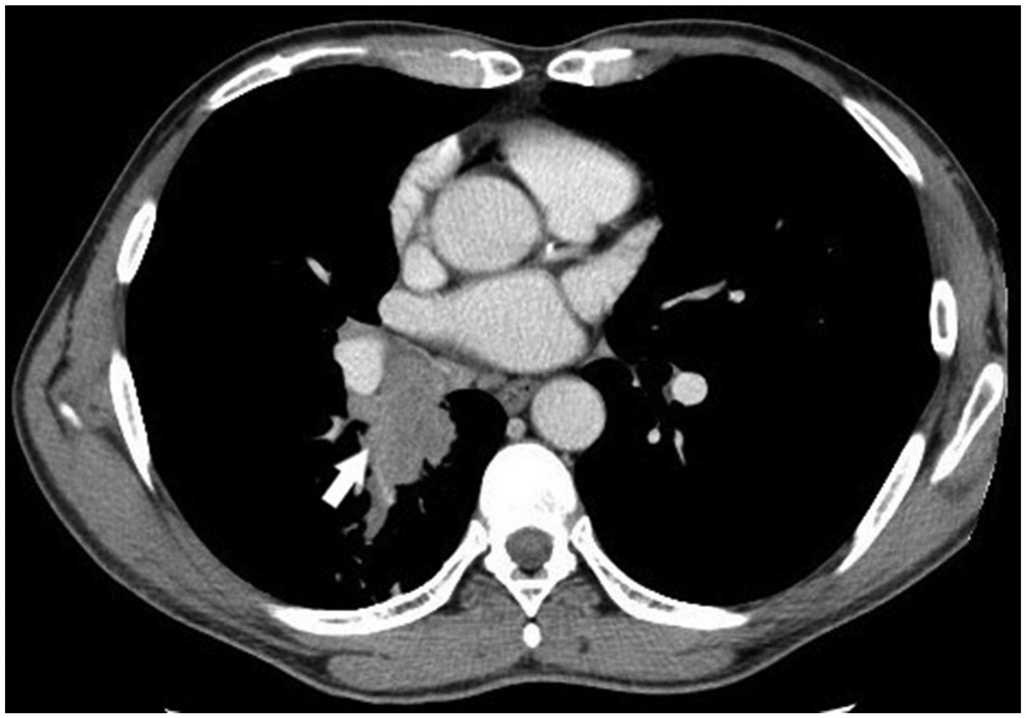
Thoracic CT scan image at the time of diagnosing SqCLC. Contrast-enhanced CT image with soft-tissue window showing a centrally located mass in the right lung with mediastinal invasion (arrow), and mediastinal lymphadenopathy.

**Figure 2.**
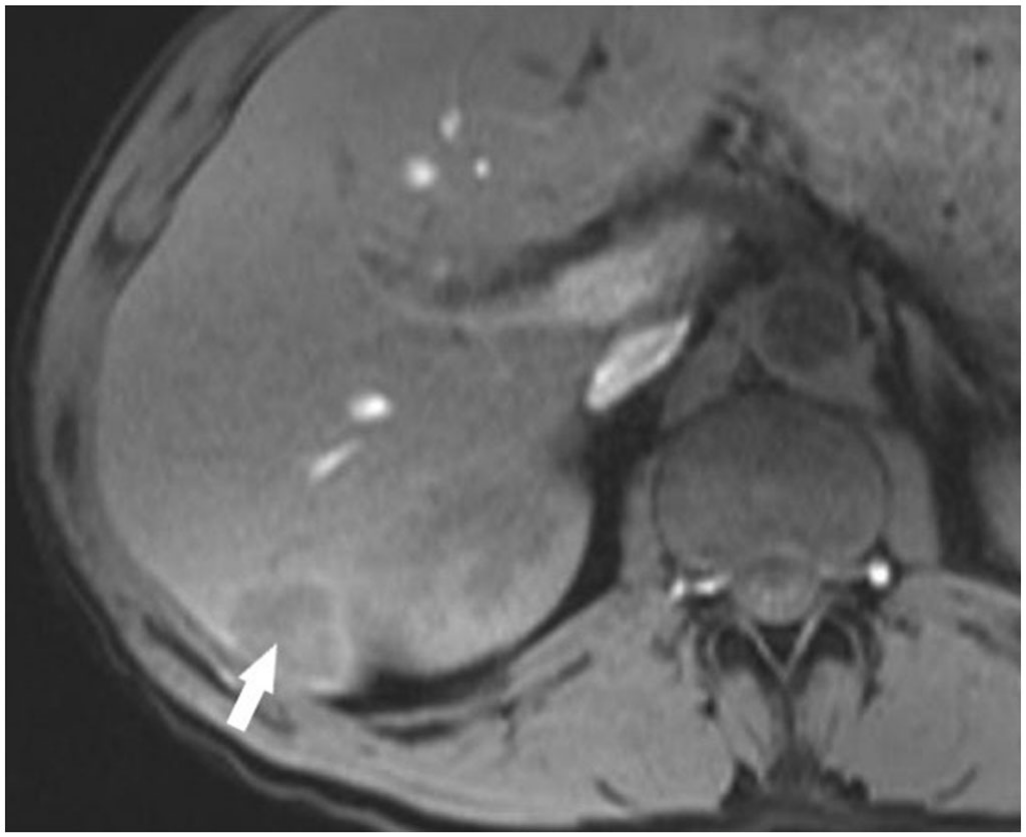
A hypovascular metastatic lesion in the liver. Liver MRI TI W image on post-contrast arterial phase showing a hypo-intense lesion with subtle concentric perilesional enhancement in segment VI of the liver (arrow).

In June 2007, a 49-year-old male suffering from chronic non-productive cough, episodic fever, and night sweats was referred to a pulmonologist. The symptoms lasted for approximately 6 months. The patient’s medical history revealed a myocardial infarction at the age of 47 years, New York Heart Association (NYHA) functional class III chronic heart failure, and smoking. Upon chest auscultation, diminished vesicular breathing sounds with crackles during forced expiration were heard in the right lung. Laboratory findings showed a slight leukocytosis (10,110/μL), elevated C-reactive protein level (40.2 mg/L) and high erythrocyte sedimentation rate (86 mm/h). A computed tomography (CT) scan was performed, which revealed a centrally located mass in the right lung, invading the mediastinum and occluding the right lower lobe bronchus. Multiple bilaterally enlarged paratracheal and subcarinal lymph nodes were also present (Figure 1). The patient underwent an endobronchial biopsy of the mass, which led to the diagnosis of SqCLC. Furthermore, a 2.6 × 2.3 × 2.0 cm hypovascular lesion in segment VI of the liver was detected in an abdominal CT scan. Although no biopsy of the lesion was performed, it was considered as a possible metastasis based on gadolinium-enhanced magnetic resonance imaging (MRI) scan results (Figure 2). Therefore, the SqCLC was clinically staged as cT4N3M1 (stage IV).

**Figure 3.**
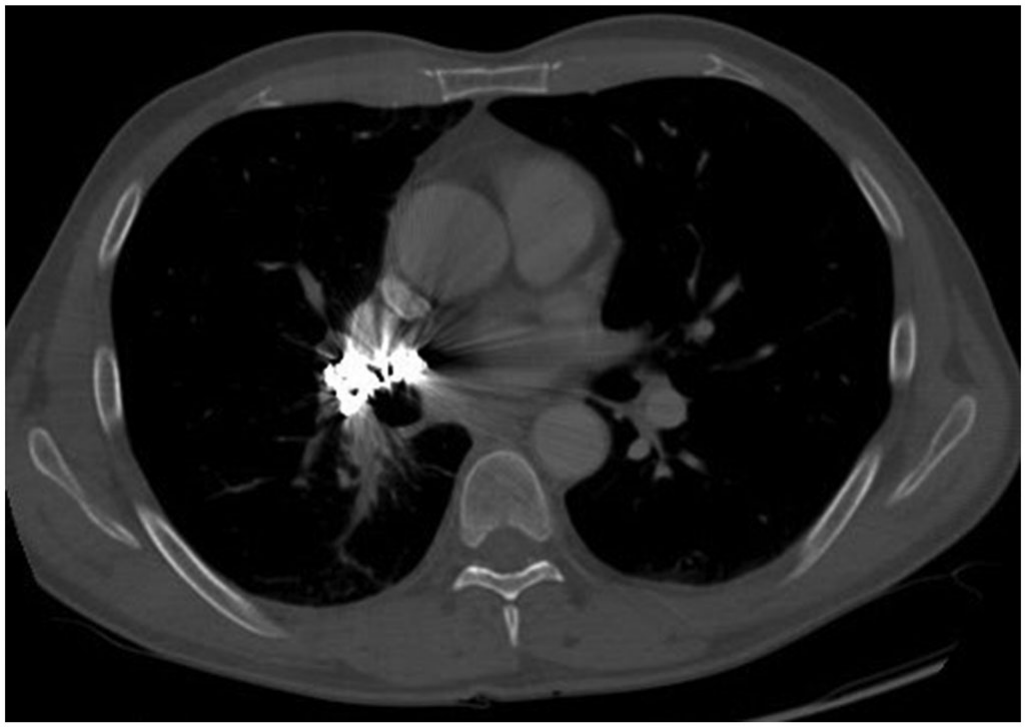
Thoracic CT scan image after the pulmonary artery embolisation procedure. Contrast-enhanced CT image with soft-tissue window showing multiple coils embolised to the right main pulmonary artery, as well as a partially decreased tumour and reduced occlusion of the right lower lobe bronchus.

Since the disease was diagnosed at an advanced stage, the patient was not a candidate for pneumonectomy and was scheduled for chemotherapy treatment instead. However, the initiation of chemotherapy was delayed as the patient experienced massive haemoptysis soon after being diagnosed with SqCLC. The pulmonary haemorrhage was managed by obturating the intermediate bronchus and performing CT pulmonary angiography and coil embolisation of the right pulmonary artery in June 2007 (Figure 3). A total of 33 Gianturco 3–8 mm diameter coils were used for the embolisation, allowing only minimal residual blood flow to the right lung. Two weeks later, the bronchial obturator was removed and the haemorrhage did not recur after the procedure.

**Figure 4.**
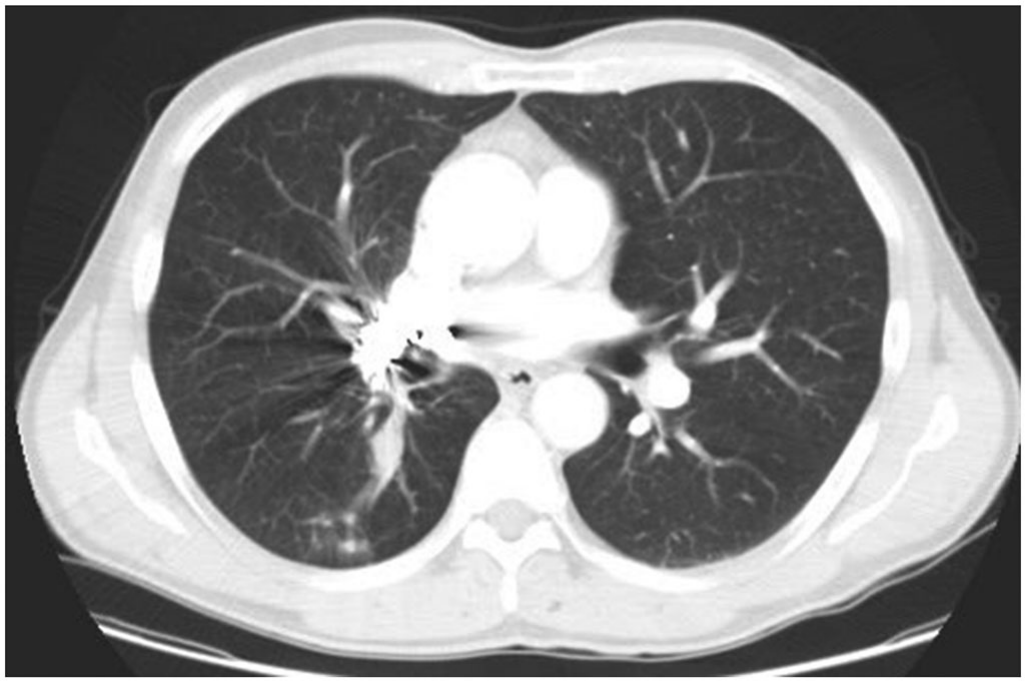
Thoracic CT scan image in 2009. A follow-up contrast-enhanced CT image with soft-tissue window after 2 months of treatment with second-line chemotherapy showing an increased tumour in the right lung.

In July 2007, first-line chemotherapy with cisplatin and gemcitabine was initiated for six cycles of treatment, which resulted in primary tumour size reduction from 6.4 × 3.0 × 6.5 cm to 1.5 × 1.2 × 1.2 cm in axial, coronal, and sagittal planes, with no effect on the liver metastasis. The patient remained stable until August 2009, when increased lung tumour size (2.8 × 2.2 cm) was observed in a follow-up CT scan and the disease was termed as progressive according to the response evaluation criteria in solid tumours (RECIST). At this point, no rebiopsy was performed. The patient was then included in a clinical trial and received six cycles of second-line chemotherapy with docetaxel and either a tyrosine kinase inhibitor nintedanib or a placebo. It is unknown whether the patient was in the treatment or in the control group. Despite the chemotherapy, the disease appeared to progress further as the primary tumour continued to grow in size based on evaluation of the CT scans (Figure 4). In contrast, the metastatic lesion in the liver has no longer been visible on ultrasound or CT imaging since 2011, which could be regarded as a mixed response to the treatment.

In 2010, 2011, and 2019, three biopsies of the lung tumour were performed due to radiological changes suggestive of SqCLC progression, i.e., occurrence of several new pulmonary lesions and increased primary tumour volume. However, only excess connective tissue formation with no SqCLC cells was observed in each of the tissue samples. Since the biopsies have been non-informative, a whole-body ^18^F-fluorodeoxyglucose (^18^FDG) positron emission tomography-CT (PET-CT) scan was performed in January 2020 in order to exclude disease progression. The only notable finding in the ^18^FDG PET-CT scan was an area of low metabolic activity among the coils, which can be considered physiological. No pathological metabolic activity in the liver or elsewhere in the body was detected. Due to the lack of evidence indicating cancer progression, the patient has not received treatment for SqCLC since 2009. Currently, the patient remains in a stable clinical condition with a good performance status—0 on the Eastern Cooperative Oncology Group (ECOG) scale.

## Discussion

We have demonstrated a rare case of spontaneous regression of advanced stage SqCLC in a patient who has already survived for nearly 13 years since receiving the diagnosis. Spontaneous regression is frequently defined as the ‘partial or complete disappearance of a malignant tumour in the absence of treatment or in the presence of therapy considered inadequate to exert a significant influence on the disease’ in cases when the diagnosis of cancer is proven by histopathological examination in the first place (10,11). According to the widely accepted criteria formulated by Everson and Cole, the term can be applied to both the regression of the primary tumour as well as its metastases, the presence of which can be either confirmed histopathologically or suspected using radiological imaging (8,10). In our patient’s case, it appears that the phenomenon of spontaneous regression occurred both in the primary tumour and the metastatic lesion of the liver. It is important to note that the diagnosis of metastatic disease was established solely on the basis of hepatic lesion appearance in the MRI scan. At the time, a PET scan was not available in our country for detection of metastatic lesions, and no biopsy of the suspected liver metastasis was taken. Nevertheless, according to a meta-analysis of 10 studies, gadolinium-enhanced MRI scan is an excellent diagnostic tool for metastatic lesions in the liver. The pooled specificity of gadolinium-enhanced MRI and ^18^FDG PET-CT is 0.99 and 1.00, respectively (12). Hence, only a minuscule possibility remains that the hepatic lesion in our patient was an unrelated finding, such as a haemangioma or an area of liver steatosis.

The most prevalent explanations for spontaneous lung cancer regression involve activation of the immune system. For instance, it has been observed that the phenomenon often occurs soon after acute infections, which led some authors to believe that the activation of innate immunity is responsible for the disease regression (11). Although the exact biological mechanism of this effect is unknown, it is thought that immunological response results in either T-cell-mediated apoptosis or inflammatory necrosis of the tissues (13,14). However, infection-related immune response in our patient is unlikely as there was no evidence of significant infections during the observation period. Tumour regression has also been observed after performing invasive procedures, such as a biopsy or surgery of the neoplasm, possibly due to the release of antigens into the bloodstream and subsequent activation of the immune response (15,16). Another theory involving the immune system is based on the psychoneuroimmunological mechanisms, suggesting that positive psychological changes can stimulate the cellular immune response and may thus induce cancer regression in some patients (13,17,18). Naturally, it is extremely difficult to prove or disprove this mechanism being responsible for disease regression in any given specific case, including the one we describe.

We believe that in our case cancer regression was induced by performing endovascular embolisation of the right pulmonary artery with 33 coils due to pulmonary haemorrhage. We speculate that coil embolisation resulted in locally impaired angiogenesis, which led to primary tumour cell death and prevented further progression. When the tumour cells become deprived of oxygen and nutrients, they produce angiogenic growth factors which bind to the receptors located in the endothelium of pre-existing blood vessels. This results in morphological changes of the original vessels and formation of new ones during a multi-step process (19,20). However, in our patient’s case, the vascular supply of the neoplasm became inadequate after coil embolisation as the blood flow via the main tumour-feeding artery was drastically reduced. Therefore, both the blood supply and angiogenic potential of the tumour could have been impaired by this procedure. Upon review of the literature, we were unable to find any similar cases attributing primary lung cancer regression to endovascular embolisation of tumour-feeding arteries. To our knowledge, only metastatic pulmonary lesions from primary renal cell carcinoma as well as some benign thoracic tumours have been reported to regress following transarterial embolisation (21). Furthermore, the disappearance of our patient’s metastatic lesion in the liver is also obscure. It has been previously suggested that the removal of the primary tumour may provoke an immune response which can eliminate metastatic lesions (22). It is possible that a similar mechanism may have been triggered by the coil embolisation procedure and subsequent regression of the primary tumour. Nevertheless, similarly to the other suggested possible underlying mechanisms of cancer regression, these theories are only hypothetical and require extensive further research to be confirmed.

It should also be taken into account that our patient has received two courses of chemotherapy treatment. The first-line treatment with cisplatin and gemcitabine appeared to be rather effective in reducing the primary tumour size. However, the initiation of chemotherapy coincided with the endovascular embolisation procedure, which could have also led to tumour volume reduction (21). Even if the initial radiological improvement can be attributed to systemic treatment, according to the literature, the chemotherapy regimens used for treating our patient are highly unlikely to induce a long-term remission. For instance, the median survival of advanced-stage SqCLC patients treated with cisplatin and gemcitabine is only 10.8 months. Moreover, the median progression-free survival is 5.1 months, and the survival rate at 24 months post-treatment is 14% (23). Regarding second-line chemotherapy, the median survival of patients treated with docetaxel is 6 months, while the median progression-free survival is only 2.8 months (24). It is unknown whether a tyrosine kinase inhibitor was added to our patient’s treatment regimen along with docetaxel as he received the treatment in a clinical trial setting. Either way, the radiological appearance of the primary tumour did not improve after six cycles of second-line chemotherapy. Thus, it seems improbable that the systemic treatment led to cancer remission in our patient’s case.

## Conclusions

In this paper, we present a case of long-term SqCLC remission following the procedure of pulmonary artery coil embolisation. We suggest that coil embolisation of tumour-feeding arteries should be considered as a potential treatment method in patients with SqCLC and other histological types of NSCLC. However, further studies are needed to support our proposition and elucidate the molecular mechanisms behind cases of spontaneous cancer regression.

## Consent for Publication

Written consent was obtained from the patient for publication of this case report and for the use of accompanying images. The authors have fully anonymized the patient.

## References

[1] Herbst RS, Morgensztern D, Boshoff C The biology and management of non-small cell lung cancer. Nature 2018;553:446–54. doi:10.1038/nature25183 29364287

[2] Lu T, Yang X, Huang Y, Zhao M, Li M, Ma K, et al. Trends in the incidence, treatment, and survival of patients with lung cancer in the last four decades. CMAR. 2019;11:943–53. doi:10.2147/CMAR.S187317 PMC6345192 30718965

[3] Zappa C, Mousa SA Non-small cell lung cancer: current treatment and future advances. Transl Lung Cancer Res. 2016;5:288–300. doi:10.21037/tlcr.2016.06.07 27413711 PMC4931124

[4] Kenfield SA, Wei EK, Stampfer MJ, Rosner BA, Colditz GA Comparison of aspects of smoking among four histologic types of lung cancer. Tob Control. 2008;17:198–204. doi:10.1136/tc.2007.022582 18390646 PMC3044470

[5] Roth JA, Goulart BHL, Ravelo A, Kolkey H, Ramsey SD Survival gains from first‐line systemic therapy in metastatic non‐small cell lung cancer in the U.S., 1990–2015: progress and opportunities. The Oncol. 2017;22:304–10. doi:10.1634/theoncologist.2016-0253 PMC5344635 28242792

[6] Siegel RL, Miller KD, Jemal A Cancer statistics, 2018. CA Cancer J Clin. 2018;68:7–30. doi:10.3322/caac.21442 29313949

[7] Suh Y-G, Cho J Local ablative radiotherapy for oligometastatic non-small cell lung cancer. Radiat Oncol J. 2019;37:149–55. doi:10.3857/roj.2019.00514 31591862 PMC6790793

[8] Ariza ‐Prota M, Martínez C, Casan P Spontaneous regression of metastatic squamous cell lung cancer. Clin Case Rep. 2018;6:995–8. doi:10.1002/ccr3.1502 29881550 PMC5986011

[9] Chung C, Park DI, Kim SY, Kim JO, Jung SS, Park HS, et al. Spontaneous regression of non-small cell lung cancer that progressed after multiple chemotherapies: a case report. Thorac Cancer. 2015;6:805–7. doi:10.1111/1759-7714.12221 26557923 PMC4632937

[10] Everson TC Spontaneous regression of cancer. Prog Clin Cancer 1967;3:79–95. 4867093

[11] Jessy T Immunity over inability: the spontaneous regression of cancer. J Nat Sci Biol Med. 2011;2:43–9. doi:10.4103/0976-9668.82318 22470233 PMC3312698

[12] Deng J, Tang J, Shen N Meta-analysis of diagnosis of liver metastatic cancers: comparison of 18FDG PET-CT and gadolinium-enhanced MRI. J Med Imaging Radiat Oncol. 2014;58:532–7. doi:10.1111/1754-9485.12231 25208683

[13] Gladwish A, Clarke K, Bezjak A Spontaneous regression in advanced non-small cell lung cancer. BMJ Case Rep. 2010;2010:bcr0720103147. doi:10.1136/bcr.07.2010.3147 PMC3029413 22802473

[14] Tadmor T Time to understand more about spontaneous regression of cancer. Acta Haematol. 2019;141:156–7. doi:10.1159/000496680 30799410

[15] Ogawa R, Watanabe H, Yazaki K, Fujita K, Tsunoda Y, Nakazawa K, et al. Lung cancer with spontaneous regression of primary and metastatic sites: a case report. Oncol Lett. 2015;10:550–2. doi:10.3892/ol.2015.3243 26171067 PMC4487128

[16] Lopez-Pastorini A, Plönes T, Brockmann M, Ludwig C, Beckers F, Stoelben E Spontaneous regression of non-small cell lung cancer after biopsy of a mediastinal lymph node metastasis: a case report. J Med Case Reports. 2015;9:217. doi:10.1186/s13256-015-0702-9 PMC4573999 26377170

[17] Ooi KH, Cheo T, Soon GST, Leong CN Spontaneous regression of locally advanced nonsmall cell lung cancer. Medicine (Baltimore). 2018;97:e11291. doi:10.1097/MD.0000000000011291 30075496 PMC6081199

[18] Kiecolt-Glaser JK, Robles TF, Heffner KL, Loving TJ, Glaser R Psycho-oncology and cancer: psychoneuroimmunology and cancer. Ann Oncol. 2002;13:165–9. doi:10.1093/annonc/mdf655 12401684

[19] Jászai J, Schmidt M Trends and challenges in tumor anti-angiogenic therapies. Cells 2019;8:pii:1102. doi:10.3390/cells8091102 PMC6770676 31540455

[20] Niu G, Chen X PET imaging of angiogenesis. PET Clin. 2009;4:17–38. doi:10.1016/j.cpet.2009.04.011 20046926 PMC2753532

[21] Lorenz JM, Navuluri R Embolization of chest neoplasms: the next frontier in interventional oncology? Semin Intervent Radiol. 2019;36:176–82. doi:10.1055/s-0039-1692658 31435125 PMC6699877

[22] Kumar T, Patel N, Talwar A Spontaneous regression of thoracic malignancies. Respir Med. 2010;104:1543–50. doi:10.1016/j.rmed.2010.04.026 20580882

[23] Scagliotti GV, Parikh P, von Pawel J, Biesma B, Vansteenkiste J, Manegold C, et al. Phase III study comparing cisplatin plus gemcitabine with cisplatin plus pemetrexed in chemotherapy-naive patients with advanced-stage non–small-cell lung cancer. JCO. 2008;26:3543–51. doi:10.1200/JCO.2007.15.0375 18506025

[24] Brahmer J, Reckamp KL, Baas P, Crinò L, Eberhardt WEE, Poddubskaya E, et al. Nivolumab versus docetaxel in advanced squamous-cell non–small-cell lung cancer. N Engl J Med. 2015;373:123–35. doi:10.1056/NEJMoa1504627 26028407 PMC4681400

